# Neuropathic Pruritus as a Precursor to Delusional Parasitosis in Multiple Sclerosis: A Proposed Clinical Trajectory and Case Report

**DOI:** 10.7759/cureus.96435

**Published:** 2025-11-09

**Authors:** Shannon Weatherly, Austin Chen, Laura Ziton

**Affiliations:** 1 Psychiatry, Nova Southeastern University Dr. Kiran C. Patel College of Osteopathic Medicine, Fort Lauderdale, USA; 2 Family Medicine, Nova Southeastern University Dr. Kiran C. Patel College of Osteopathic Medicine, Fort Lauderdale, USA; 3 Family Medicine, Northwest Medical Center, Margate, USA; 4 Family Medicine, Broward Health, Coral Springs, USA; 5 Family Medicine, Dr. Kiran C. Patel College of Osteopathic Medicine, Fort Lauderdale, USA

**Keywords:** aripiprazole, delusional parasitosis, multiple sclerosis, neuropathic pruritus, somatic delusion

## Abstract

Multiple sclerosis (MS) is a chronic demyelinating disorder of the central nervous system that is associated with a range of neuropsychiatric complications, including depression, anxiety, and cognitive impairment. Delusional parasitosis is a rare and underrecognized manifestation in patients with MS. It is characterized by a fixed false belief of infestation with parasites or other organisms despite the absence of supporting medical evidence. In some cases, the condition may be preceded by chronic neuropathic pruritus, which can contribute to the development of somatic delusions.

A 76-year-old woman with a longstanding history of MS developed persistent generalized pruritus and underwent multiple dermatologic evaluations, all of which yielded negative results. Despite the absence of objective findings, she received numerous courses of antiparasitic and anti-inflammatory treatments over three years. Her symptoms progressed to a fixed belief of infestation, which persisted despite repeated disconfirmation. The condition was associated with substantial psychosocial consequences, including isolation and the dissolution of long-term relationships. This case illustrates a possible clinical progression from neuropathic pruritus to delusional parasitosis in MS. The findings underscore the importance of early recognition to potentially prevent progression, as well as the need for coordinated care involving primary care, neurology, dermatology, and psychiatry.

## Introduction

Multiple sclerosis (MS) is a chronic demyelinating disorder of the central nervous system that is frequently associated with neuropsychiatric complications, including depression, anxiety, cognitive impairment, and, less commonly, psychosis. One of the rarest neuropsychiatric presentations in MS is delusional parasitosis, also known as Ekbom syndrome. This condition is classified as a somatic subtype of delusional disorder and is characterized by a fixed false belief of infestation with parasites, insects, or other organisms despite the absence of medical evidence.

According to the Diagnostic and Statistical Manual of Mental Disorders, Fifth Edition (DSM-5), delusional disorder, somatic type, is defined by one or more persistent non-bizarre delusions lasting at least one month, without meeting the criteria for schizophrenia, and with relatively preserved overall functioning [1]. In the somatic subtype, the central delusion involves bodily functions or sensations, which are often misinterpreted as signs of illness or infestation. Patients commonly report abnormal cutaneous sensations such as crawling, biting, or stinging, which they attribute to infestation. These symptoms frequently lead to compulsive skin-picking, excoriations, and significant psychosocial distress. Delusional parasitosis may present as a primary psychiatric disorder or as a secondary phenomenon associated with neurologic diseases, systemic illnesses, or medication side effects. In MS, neuropathic pruritus is a well-recognized sensory symptom that is typically paroxysmal and associated with demyelinating lesions in the central nervous system [2]. Although neuropathic pruritus is a sensory phenomenon, in susceptible individuals, it may act as both a trigger and a reinforcing factor for the development of somatic delusions [3-4]. This progression represents a critical clinical transition in which a legitimate neurologic symptom becomes incorporated into a fixed delusional framework.

## Case presentation

A retrospective chart review was conducted for a single patient with MS and neuropathic pruritus treated in a primary care setting between 2022 and 2025. Records from primary care, dermatology, neurology, allergy, and emergency department visits were reviewed. The patient was a 76-year-old woman with longstanding MS who developed generalized pruritus in April 2022. Examination at that time documented non-draining papules with excoriations on the extremities and scalp. Vital signs were within normal limits, and there was no evidence of drainage, ulceration, or infection. No ear, nasal, or mucosal abnormalities were noted. She denied any recent travel or new sexual partners and reported fatigue, sleep disturbance, and social withdrawal related to the itching. She was referred to dermatology for further evaluation.

Baseline MS status was not documented, and disease activity was not discussed with the patient. Baseline laboratory values and medication history were unavailable, and she was not actively engaged in neurological care at the time of presentation. Her chart history noted intermittent prednisone use for presumed MS flare-ups. There were no prior episodes of neuropsychiatric symptoms or delusional behavior known before the onset of pruritus. Over the next year, dermatologic evaluations, including biopsies and infectious testing, were negative. A trial of gabapentin for presumed neuropathic pruritus was initiated but failed to provide symptomatic relief. By May 2023, she developed a fixed belief that she was infested with scabies, which persisted despite reassurance and negative findings. Although a psychiatric evaluation was recommended, the patient declined referral.

From mid-2023 through early 2025, she received multiple topical, oral, and biologic therapies, including corticosteroids, tacrolimus, doxycycline, permethrin, ivermectin, and dupilumab, without improvement. During this period, she continued to seek medical care across specialties, including several emergency department visits where she insisted she remained infested with parasites and requested ivermectin. Physical examination consistently revealed diffuse excoriations without new lesions or systemic abnormalities. Her pruritus was accompanied by worsening fatigue, disrupted sleep, and increasing social isolation, including dissolution of a long-term relationship.

At a June 2025 primary care visit, microscopy of a specimen she identified as a parasite revealed no evidence of infestation. Despite these findings, her fixed belief persisted. She again declined psychiatric referral but consented to initiate aripiprazole 5 mg daily as a potential solution for her itching, despite limited insight into her delusional preoccupation. At the follow-up, she reported partial improvement in itching; however, medication adherence remains uncertain as she has since missed two scheduled follow-up visits.

## Discussion

Delusional parasitosis is a rare but important neuropsychiatric manifestation in MS. This case highlights how neuropathic pruritus, a recognized sensory symptom of MS, can contribute to the development of somatic delusions in susceptible individuals. Chronic pruritus may act as both a trigger and a reinforcing factor for fixed delusional beliefs, particularly in the presence of disease-related cortical dysfunction, psychiatric comorbidities, or psychosocial stressors.

Neuropathic itch arises from damage to neurons in the central or peripheral nervous system, disrupting itch-signaling pathways and producing spontaneous neuronal firing with altered sensory processing [5]. It may develop in the context of metabolic, neurodegenerative, orthopedic, infectious, autoimmune, malignant, or iatrogenic conditions affecting the somatosensory system [5]. Clinically, it can resemble a modality-specific sensory hallucination, similar to phantom limb pain or phantom noise tinnitus. Diagnosis is often challenging and requires a detailed history, targeted physical examination, and exclusion of alternative causes.

In multiple MS, paroxysmal neuropathic pruritus is frequently associated with demyelinating lesions in the cervical spinal cord and brainstem [2]. Such lesions promote spontaneous activity in primary afferent pruriceptive neurons, central sensitization within dorsal horn neurons, and impaired sensory integration across ascending pathways [6]. Central sensitization can allow normally innocuous stimuli to activate pruriceptive networks, producing allokinesis and hyperkinesis. Disrupted inhibitory modulation between pain and itch pathways may further sustain somatic misinterpretation and reinforce delusional ideation [6].

**Figure 1:**
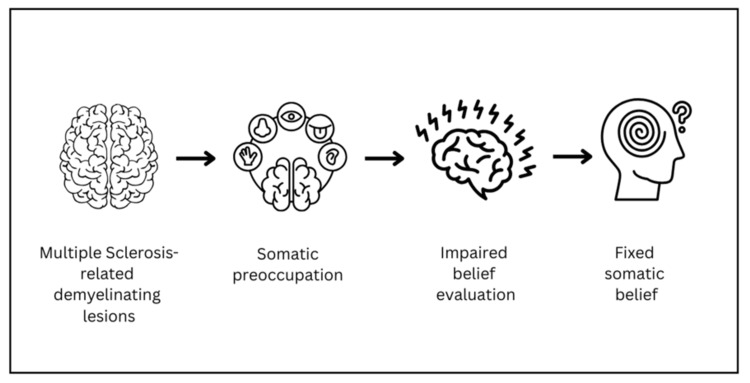
Proposed mechanism linking multiple sclerosis-related neuropathic pruritus to delusional parasitosis

The two-factor model of monothematic delusions offers a framework for progression from neuropathic pruritus to delusional parasitosis. The first factor involves abnormal sensory input: in this case, neuropathic pruritus driven by multiple sclerosis-related demyelination and neural injury. The second factor is impaired belief evaluation, which may be influenced by MS-associated cortical dysfunction, psychiatric comorbidity, or psychosocial stressors [7-8]. White matter lesions in periventricular, cerebellar, and brainstem regions have been associated with psychosis in MS, supporting the role of structural changes in disrupting cognitive and emotional regulation [8-9]. Dopaminergic dysfunction, including reduced dopamine transporter activity, has also been implicated in primary and secondary delusional parasitosis, providing a mechanistic rationale for antipsychotic therapy [10]. Demyelinating lesions may trigger neuropathic pruritus and subsequent somatic preoccupation, as illustrated in Figure 1, which outlines the proposed mechanism linking MS-related neuropathic pruritus to delusional parasitosis.

In vulnerable individuals, impaired belief evaluation, due to cortical dysfunction, psychiatric comorbidity, or social stress, can lead to fixed somatic delusions. This model reflects the two-factor theory of delusion formation [7].

**Figure 2:**
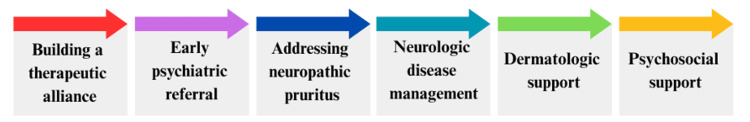
Proposed multidisciplinary management approach for delusional parasitosis in multiple sclerosis

Management requires early recognition and coordinated intervention. A proposed multidisciplinary approach integrates psychiatric, neurologic, dermatologic, and psychosocial domains (Figure 2). Key steps include building a therapeutic alliance, early psychiatric referral, treatment of neuropathic pruritus, optimization of MS disease management, dermatologic support, and psychosocial interventions. Establishing trust is critical, as patients may resist psychiatric involvement due to stigma or the fixed nature of their beliefs [4]. Second-generation antipsychotics, including aripiprazole, risperidone, and olanzapine, have demonstrated efficacy in reported cases [11]. Aripiprazole may be particularly useful due to its combined dopaminergic and serotonergic activity, favorable tolerability, and observed response within six weeks [11-12]. As a partial D₂ and 5-HT₁A agonist with 5-HT₂A antagonism, it helps stabilize dopaminergic pathways implicated in delusional thinking while modulating serotonergic circuits involved in itch perception. This dual mechanism may alleviate both the neuropathic pruritus and the accompanying somatic delusion observed in this patient population [12].

Concurrent treatment of neuropathic pruritus is essential, as ongoing somatic sensations can reinforce delusional ideation. First-line pharmacologic options include gabapentin, with refractory cases potentially benefiting from topical agents such as capsaicin or compounded amitriptyline/ketamine/lidocaine [2-3]. Our patient received a trial of gabapentin without symptomatic relief, prompting further evaluation and eventual treatment with aripiprazole for delusional parasitosis secondary to neuropathic pruritus. Neurologic follow-up is important for optimizing disease-modifying therapy and monitoring lesion progression that could exacerbate neuropsychiatric symptoms [9]. Dermatologic care aids wound management and helps confirm the absence of parasitic pathology, reducing diagnostic uncertainty and supporting psychiatric acceptance. Psychosocial interventions, including cognitive-behavioral therapy, habit reversal training, and caregiver education, can further help disrupt the itch-scratch-delusion cycle [3-4].

A comprehensive, interdisciplinary approach that addresses both the neuropsychiatric and neurologic components offers the greatest potential for symptom resolution and improved outcomes in MS-associated delusional parasitosis.

## Conclusions

This report highlights a plausible clinical trajectory in which neuropathic pruritus in MS evolves into delusional parasitosis. This phenomenon may represent not only a neuropsychiatric complication but also a potential marker of disease progression. The observed sequence, from neurologically mediated sensory disturbance to somatic preoccupation and ultimately to fixed delusion, underscores the complex interplay between demyelinating pathology, altered sensory processing, and impaired belief evaluation. Recognizing this progression at its earliest stages may enable timely psychiatric intervention, reduce unnecessary diagnostic and therapeutic interventions, and optimize multidisciplinary management. Greater awareness of this potential pathway could refine clinical vigilance and prompt the development of targeted strategies for prevention and early treatment.
